# SWIM (sickle with ibuprofen and morphine) randomised controlled trial fails to recruit: lessons learnt

**DOI:** 10.1136/bmjopen-2016-011276

**Published:** 2016-06-09

**Authors:** Gavin Cho, Kofi A Anie, Jacky Buckton, Patricia Kiilu, Mark Layton, Lydia Alexander, Claire Hemmaway, Dorothy Sutton, Claire Amos, Caroline J Doré, Brennan Kahan, Sarah Meredith

**Affiliations:** 1 Haematology and Sickle Cell Centre, London North West Healthcare NHS Trust, Central Middlesex Hospital, London, UK; 2 Faculty of Medicine, Imperial College London, London, UK; 3 Department of Haematology, Imperial College London, Hammersmith Hospital Campus, London, UK; 4 Department of Haematology, Imperial College Healthcare NHS Trust, Hammersmith Hospital, London, UK; 5 Department of Haematology, Barking, Havering and Redbridge University Hospitals NHS Trust, Queen's Hospital, Romford, Essex, UK; 6 MRC Clinical Trials Unit, University College London, London, UK

**Keywords:** PAIN MANAGEMENT, ACCIDENT & EMERGENCY MEDICINE, SICKLE CELL DISEASE, OPIOIDS, IBUPROFEN

## Abstract

**Objectives:**

Sickle With Ibuprofen and Morphine (SWIM) trial was designed to assess whether co-administration of ibuprofen (a non-steroidal anti-inflammatory drug) resulted in a reduction of opioid consumption delivered by patient-controlled analgesia (PCA) for acute pain in sickle cell disease.

**Design:**

A randomised, placebo-controlled, double-blind trial.

**Setting:**

UK multicentre trial in acute hospital setting.

**Participants:**

Adults with sickle cell disease of any gender and phenotype aged 16 years and over.

**Interventions:**

Oral ibuprofen at a dose of 800 mg three times daily or placebo in addition to opioids (morphine or diamorphine) administered via PCA pump for up to 4 days.

**Main outcome measures:**

The primary outcome measure was opioid consumption over 4 days following randomisation.

**Results:**

The SWIM trial closed early because it failed to randomise to its target of 316 patients within a reasonable time.

**Conclusions:**

The key issues identified include the unanticipated length of time between informed consent and randomisation, difficulties in randomisation of patients in busy emergency departments, availability of trained staff at weekends and out of hours, fewer centres than expected using PCA routinely for sickle cell pain treatment, lack of research staff and support for participation, and the trial design. There are implications for future UK trials in sickle cell disease.

**Trial registration number:**

ISRCTN97241637, NCT00880373; Pre-results.

**Strengths and limitations of this study**

- The SWIM trial was designed as a randomised, placebo-controlled, double-blind trial.
- SWIM failed to achieve its target rate of patient randomisation.
- The implications for future UK sickle cell trials are discussed.

## Background

Sickle cell disease comprises a group of genetic blood disorders that affect over 13 000 people in the UK predominantly of African, Caribbean, Asian, Arabian and Mediterranean origin. The hallmark symptom is pain. Over 50% of patients with sickle cell disease admitted to hospital in the UK have acute pain,^1^ commonly treated with opioids^2^ with unpleasant side effects including nausea, constipation, itching, sedation and emotional changes.

Non-steroidal anti-inflammatory drugs (NSAIDs) have been trialled in sickle cell disease and are recommended.^3^ However, a trial comparing ketoprofen with placebo plus syringe pump-administered morphine in sickle cell disease failed to demonstrate a morphine sparing effect.^4^ Ibuprofen analgesia is dose-related: a single 400 mg dose offers one in three patients with moderate-to-severe pain at least 50% relief (number-needed-to-treat (NNT) of 2.7), compared with placebo; a single 600 mg dose provides at least 50% pain relief to one in two patients (NNT of 1.7).^5^ Furthermore, patient-controlled analgesia (PCA) using morphine in sickle cell disease provides adequate pain relief with reduced opioid consumption compared with continuous infusion.^6^

## Methods

‘Sickle With Ibuprofen and Morphine’ (SWIM) trial, the first UK multicentre trial of analgesia in sickle cell disease, was a randomised, placebo-controlled, double-blind trial of ibuprofen or placebo, designed to determine whether ibuprofen could reduce PCA opioid consumption for acute sickle cell pain.

The National Research Ethics Service, and Medicines and Healthcare products Regulatory Agency approved the SWIM trial.

### Participants and recruitment

Participants were adults (aged 16 years and over) with sickle cell disease of any phenotype, admitted to hospital with acute sickle cell pain for which opioids were warranted. Exclusions were contraindications to morphine, diamorphine, or ibuprofen including peptic ulcers and NSAID-induced asthma; renal dysfunction; stroke in preceding 6 weeks; pregnancy or breastfeeding.

Recruitment was in two stages:

1. Screening, informed consent and trial registration in outpatient clinics
2. Verbal assent and randomisation in Emergency Departments (A&E) on admission for sickle cell pain requiring opioid analgesia.

Sample size calculation assumed a mean opioid consumption in the control group of 33 mg (SD 43) over 4 days.^6^ To detect a 50% reduction (90% power, 5% significance) required 286 patients; the recruitment target of 316 (158 per arm) allowed for 10% attrition.

Patients were randomised (1:1) to oral ibuprofen 800 mg three times daily, or matching placebo, in addition to morphine or diamorphine via PCA for a maximum of 4 days during hospitalisation. Randomisation used permuted blocks stratified by centre; each patient was randomised only once by assigning the patient to the next available treatment pack number with the allocation sequence generated by the MRC Clinical Trials Unit.

The primary outcome was opioid consumption over 4 days.

## Results

Daily pain and symptom scores were recorded over the 4 days (table 1). Treatment effects and 95% CIs were calculated using an unadjusted linear regression model.

**Table 1:** Clinical outcomes for each treatment arm

	Ibuprofen (n=2)	Placebo (n=5)	Difference in means (Ibuprofen vs placebo) (95% CI)
Opioid consumption over 4 days (mg)—mean (SD)	110 (45)	206 (104)	−96 (−301 to 109)
Pain score over 4 days*—mean (SD)	1.5 (0.7)	3.2 (1.4)	−1.7 (−4.4 to 1.1)
Number of self-reported side effects per patient† (mild, moderate, or severe)—mean (SD)	7.5 (0.7)	10.2 (2.2)	−2.7 (−6.9 to 1.5)
Number of self-reported side effects per patient† (severe)—mean (SD)	3.0 (1.4)	3.2 (3.1)	−0.2 (−6.3 to 5.9)

*Pain scores were measured using a 10-point scale (0–10) with higher scores indicating more pain.

†Self-reported side effects included nausea, vomiting, diarrhoea, constipation, stomach pain/discomfort, blood in stool, mood/emotional changes, sleep disturbances, dizziness, headache, itching, dry mouth, sore chest, and breathing difficulties, and each symptom was graded as none, mild, moderate, or severe.

**Figure 1:**
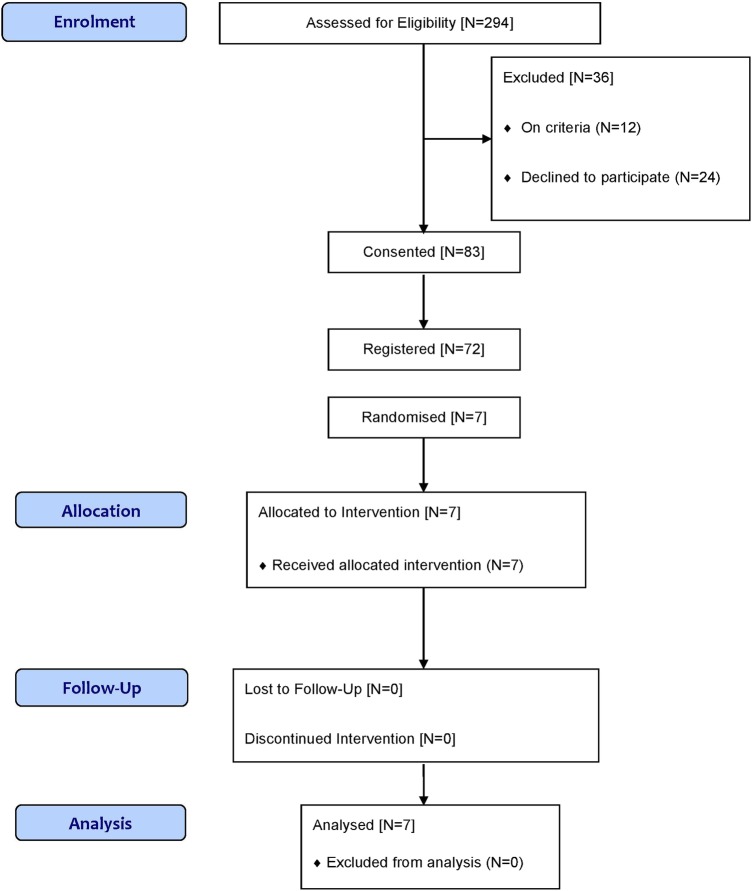
Flow chart of patient recruitment at SWIM trial closure.

The SWIM trial was terminated early by the NIHR HTA Programme due to the very slow randomisation rate. Patients were recruited over 16 months; 83 consented to the trial but only 7 patients were randomised (figure 1). Two main issues emerged at closure. First, although the number of patients giving their consent increased steadily, there was often a long delay between consent and randomisation. Patients with sickle cell disease have unpredictable pain episodes, some of which may require A&E attendances and hospital admissions. Severely affected patients tend to be offered disease-modifying treatment such as hydroxycarbamide (hydroxyurea) or blood transfusions. During the trial period, most patients who had given their consent did not have a sickle cell pain episode that required hospitalisation. One patient was admitted to another hospital which was not a trial centre at the time. Second, there was a low rate of participation by sickle cell disease treatment centres; 27 were approached, 5 did not respond, 12 declined, 10 expressed interest, 4 registered patients and only 2 centres randomised patients (table 2).

**Table 2:** Status of centre enrolment at SWIM trial closure

Centre status	Number
Recruitment started	4
Interested—ready to commence centre-specific approval	2
Interested—not ready to commence centre-specific approval	4
Declined—do not use PCA for sickle cell pain	9
Declined—staff issues, lack of research support, A&E recruitment issues	3
No response	5
Total	27

## Discussion

Several contributory factors for early closure of the SWIM trial, and potential remedies were identified:

1. Monitoring of emergency admissions for sickle cell pain at the lead trial centre found that 11 registered patients were not randomised because they presented at A&E during weekends or at night when no SWIM trial trained staff were present. Good Clinical Practice (GCP) training of A&E staff performing randomisation was challenging due to high staff turnover. A SWIM trial-specific GCP training package was developed, which was easier to deliver on a more frequent basis, but there was insufficient time for this to have an impact on randomisation rate.
2. A&E at the lead centre was closed overnight for a significant proportion of the study due to low staffing levels and safety concerns. Therefore, some registered patients were admitted to other centres. A system to allow randomisation of a registered patient admitted at a different centre was planned which would have improved the randomisation rate.
3. A SWIM trial protocol amendment to allow randomisation for repeated admissions had been approved by the trial oversight committees but not implemented before closure.^7^
4. The SWIM trial was adopted onto the National Institute for Health Research Clinical Research Network (NIHR CRN) portfolio. Nonetheless, initiation of trial centres was slow and research support was difficult to access. Several interested centres could not participate because they did not use opioid PCA. Other reasons included lack of research infrastructure and anticipated difficulties with randomisation in busy A&Es.
5. Many recruited patients with sickle cell disease did not have frequent hospitalisations for pain episodes, with a longer than anticipated delay between consent and randomisation, although it was encouraging that only 25% of eligible patients declined to participate.

The SWIM trial was conducted within the UK National Health Service (NHS) and was unsuccessful due to lack of interest or capacity at several large sickle cell disease centres, overestimation of the number of eligible patients, and unanticipated delays between registration and randomisation. USA trials in sickle cell disease also failed to recruit.^8–10^ Explanations cited include complex protocol design, insufficient staff, lack of research support, time constraints of clinical staff, requirement for trained staff at weekends and out of hours, involvement of multiple departments and fewer than expected eligible or consenting patients. These reasons are similar to the SWIM trial; nonetheless, specific strategies have to be adopted in the UK which has a different health service structure and no strong culture of sickle cell disease research to encourage successful participation. Moreover, in a cohort of multicentre trials funded by either the UK Medical Research Council or Health Technology Assessment Programme (HTA), only 31% of the trials achieved their original recruitment target with 53% being awarded an extension, and this did not improve over time.^11^ Some preidentified trial centres did not participate as planned, and there were delays due to various reasons including issues with local research staff and clinical arrangements, logistics and regulatory approvals although cancer trials were more successful because of the previously established National Cancer Research Network.^11^ Therefore, it appears that specialty clinical research networks such as those 30 prioritised by the National Institute of Health Research (NIHR) for clinical research networks subsequent to the earlier ones in the areas of medicines for children, stroke, diabetes and Alzheimer's disease would enhance recruitment.

There is a clinical need for research to improve treatment and outcomes in sickle cell disease within the NHS. The NIHR CRN portfolio provides funding; however, this is based on patients randomised, rather than patients giving consent and then recruited. In addition, CRN research capacity funds are usually awarded competitively based on research activity. Therefore, research inactive sickle cell disease centres are unlikely to be awarded funds for staff or capacity building to enable participation in trials such as SWIM. A case could be made for research in sickle cell disease to be affiliated to a specialty network to overcome these barriers.

Many HTA-funded trials incorporate a feasibility phase. The SWIM trial was in response to a priority commissioned funding opportunity, and no preliminary work had been done to identify potential problems in recruitment. Six monthly progress reports highlighted recruitment problems. Plans to address these included an amendment of the original trial design to allow each patient to be randomised on more than one occasion, as opposed to participating only once. This could have increased the accrual rate during the first year by an additional 13 randomisations. An extension of the trial was proposed to the HTA Board; however, this would have required additional funding, hence closure was not avoided.

These issues need to be addressed otherwise sickle cell disease trials in the UK will continue to fail.
